# Ectopic Expression of a Truncated Isoform of Hair Keratin 81 in Breast Cancer Alters Biophysical Characteristics to Promote Metastatic Propensity

**DOI:** 10.1002/advs.202300509

**Published:** 2023-11-10

**Authors:** Diane S. Kang, Aidan Moriarty, Yiru Jess Wang, Amal Thomas, Jia Hao, Bret A. Unger, Remi Klotz, Shamim Ahmmed, Yonatan Amzaleg, Stuart Martin, Siva Vanapalli, Ke Xu, Andrew Smith, Keyue Shen, Min Yu

**Affiliations:** ^1^ Department of Stem Cell Biology and Regenerative Medicine Keck School of Medicine of the University of Southern California Los Angeles CA 90033 USA; ^2^ USC Norris Comprehensive Cancer Center Keck School of Medicine of the University of Southern California Los Angeles CA 90033 USA; ^3^ Department of Pharmacology University of Maryland School of Medicine Baltimore MD 21201 USA; ^4^ Marlene and Stewart Greenebaum Comprehensive Cancer Center University of Maryland School of Medicine Baltimore MD 21201 USA; ^5^ Department of Molecular and Computational Biology USC David and Dana Dornsife College of Letters Arts and Sciences University of Southern California Los Angeles CA 90089 USA; ^6^ Department of Biomedical Engineering Viterbi School of Engineering University of Southern California Los Angeles CA 90089 USA; ^7^ Department of Chemistry University of California at Berkeley Berkeley CA 94720 USA; ^8^ Department of Chemical Engineering Texas Tech University Lubbock TX 79409 USA

**Keywords:** biophysical properties, breast cancer, circulating tumor cells, keratins, metastasis, truncated isoform

## Abstract

Keratins are an integral part of cell structure and function. Here, it is shown that ectopic expression of a truncated isoform of keratin 81 (*tKRT81*) in breast cancer is upregulated in metastatic lesions compared to primary tumors and patient‐derived circulating tumor cells, and is associated with more aggressive subtypes. *tKRT81* physically interacts with keratin 18 (*KRT18*) and leads to changes in the cytosolic keratin intermediate filament network and desmosomal plaque formation. These structural changes are associated with a softer, more elastically deformable cancer cell with enhanced adhesion and clustering ability leading to greater in vivo lung metastatic burden. This work describes a novel biomechanical mechanism by which *tKRT81* promotes metastasis, highlighting the importance of the biophysical characteristics of tumor cells.

## Graphical abstract

Herein, a novel biomechanical mechanism is described by which ectopic expression of a truncated isoform of keratin 81 (*tKRT81*) promotes breast cancer metastasis. *tKRT81* interacts with keratin 18, altering the cytosolic keratin intermediate filament network and desmosomal plaque formation, which is associated with a softer, more elastically deformable cell, enhanced adhesion and clustering ability, and greater in vivo lung metastasis.

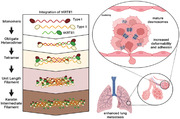

## 1 Introduction

Keratins originally gained prominence in oncology as diagnostic biomarkers for various cancer types. In breast cancer, keratin(KRT)8, KRT18, and/or KRT19 indicate luminal subtypes,^[^ ^1^ ^]^ whereas KRT5, KRT6, KRT14, and/or KRT17 are used for identifying basal subtypes.^[^ ^2^ ^]^ In addition, specific keratins are associated with certain clinicopathological tumor features and may be able to predict outcomes in patients. For these reasons, there has been a growing interest in elucidating the mechanisms by which keratin expression may promote cancer progression and metastasis.

In normal epithelial cells, the cytoplasm contains keratins that form cytoskeletal intermediate filament (IF) networks, which connect to the IFs of other cells through desmosomes, to the extracellular matrix through hemidesmosomes and provide support to the nucleus through perinuclear cages. These interconnections provide the tissue with mechanical resilience to physical forces and trauma. In general, it has been reported that one molecule of a type I keratin preferentially dimerizes with one molecule of a type II keratin in an energy‐independent manner.^[^ ^3^ ^]^ These heterodimers form tetramers, and an octamer of tetramers forms one unit length filament (ULF), which can then form the higher order filamentous structures with ≈10 nm diameter.^[^ ^3^ ^]^

In cancer cells, keratins have been linked to an invasive phenotype, high metastatic potential, and the modulation of cancer‐related signaling pathways. For example, *KRT18* has been shown to modulate estrogen receptor alpha (ER) signaling by sequestering an ER coactivator, LRP16, in the cytoplasm.^[^ ^4^ ^]^ KRT19 has been shown to help localize E‐cadherin to the cell membrane, facilitating cell‐cell adhesion.^[^ ^5^ ^]^ KRT14 is perhaps the most well‐characterized keratin in the context of cancer and has been shown to be concentrated in the leading edge of collectively invading breast cancer cells^[^ ^6^, ^7^ ^]^ and to facilitate the invasion of ovarian cancer cells.^[^ ^8^ ^]^ Keratin expression has also been linked to poor patient outcomes. Studies from two cohorts in which mRNA expression of circulating tumor cells (CTCs) from metastatic breast cancer patients were analyzed found that high expression of KRT16 is associated with a significantly shorter relapse‐free survival, with median values of 28 versus 17.5 months in one cohort and median values of 23 versus 14.8 months in another cohort for high versus low KRT16 expression, respectively.^[^ ^9^ ^]^

As CTCs are known to contain critical metastatic initiating populations,^[^ ^10^, ^11^, ^12^ ^]^ we sought to further examine the role of keratins in CTC biology and the metastatic cascade. In our previous study, we examined the metastatic potential of patient‐derived CTCs in mouse models^[^ ^13^ ^]^ and identified an upregulation of a truncated isoform of KRT81 (*tKRT81*) in lung‐metastatic CTC‐derivatives. Although expression of KRT81, a type II hair and nail keratin, has recently been shown to correlate with poor prognosis in a number of cancers,^[^ ^14^, ^15^, ^16^, ^17^, ^18^, ^19^ ^]^ distinctions regarding the full‐length and truncated isoforms of these keratins are lacking. Here, we further defined the biophysical and functional consequences of *tKRT81* upregulation in the metastatic process.

## 2 Results

### 2.1 KRT81 Is Upregulated in Metastatic Derivatives of CTCs Compared to the Isogenic, Parental CTC Lines, and Is Associated with Lung Metastasis Free Survival

**Figure 1:**
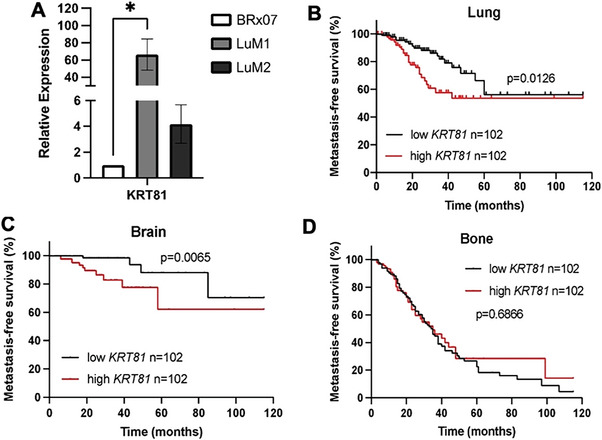
KRT81 upregulation is associated with metastasis. A) Quantitative RT‐PCR of KRT81 in 2 lung metastatic CTC derivatives (LuM1 and LuM2) compared to the isogenic parental CTC line (BRx07). Mean ± SEM, *n* = 3. *p = 0.0223, Statistical significance was calculated by unpaired *t*‐test. B–D) Kaplan–Meier survival curves of lung (B) brain (C) and bone (D) metastasis‐free survival in a publicly available dataset, GSE12276. Significance was calculated by log‐rank test.

Our previous study characterizing the metastatic tropism of ex vivo cultured patient‐derived CTC lines (referred to as BRx07, BRx68, BRx50, and BRx42), which displayed strikingly similar tropism to the actual metastases of the patients from whom the CTCs were derived,^[^ ^13^ ^]^ generated RNA sequencing data for lung, brain, bone, and ovary metastases arising from each given isogenic, matched parental CTC line. Differential RNA sequencing analyses revealed *KRT81* as significantly upregulated in metastatic sites compared to matched parental CTC lines (**Table** **1**). *KRT81* upregulation in lung‐metastatic CTC derivatives of BRx07 (referred to as LuM1 and LuM2) compared to the parental, isogenic CTC line (BRx07) was confirmed by qPCR using primers detecting the 3′‐end of the gene (**Figure** **1**A). Importantly, analysis of the GSE12276 cohort of breast cancer patients, where 204 women were followed for sites of metastatic relapse, showed that higher *KRT81* expression levels in the primary tumor were significantly associated with a shorter lung and brain metastasis‐free survival (Figure 1B,C, respectively) but not bone metastasis‐free survival (Figure 1D), further indicating the relevance of our CTC derived data and demonstrating the significance of KRT81 in metastasis.

**Table 1:** Differential gene expression analyses from RNA‐seq data identify significant upregulation of KRT81 expression in various metastatic derivatives (FDR cutoff 0.1).

Comparison	p‐adjusted	Fold change
BRx07 Lung vs BRx07 CTCs	2.68 × 10^−4^	25.8
BRx07 Ovary vs BRx07 CTCs	5.09 × 10^−5^	35.5
BRx07 All Mets vs BRx07 CTCs	1.36 × 10^−5^	30.7
All Mets vs All CTCs	4.68 × 10^−5^	10.1
All Lung Mets vs All Other Mets	6.87 × 10^−3^	16.0
BRx68 Bone vs BRx68 CTCs	1.70 × 10^−4^	27.9

### 2.2 Identification of Truncated KRT81 (*tKRT81*) in Breast Cancer Cell Lines and Patient Samples

**Figure 2:**
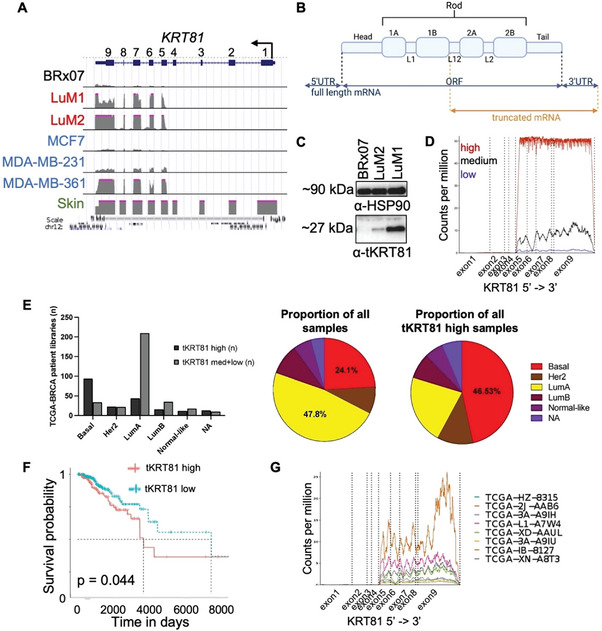
Identification of a truncated isoform of KRT81 in breast cancer cell lines and patient samples. A) Genome browser map of RNA sequencing reads from BRx07 CTC line (black), isogenic lung‐metastatic BRx07 CTC derivatives (red), and publicly available data from commonly used breast cancer cell lines (blue) aligned to the KRT81 gene. Data from the skin (green), where full‐length KRT81 is normally expressed, is shown for comparison. B) A schematic depicting the protein domains encoded by full‐length KRT81 mRNA transcript compared to that of truncated KRT81. C) Immunoblot shows a band corresponding to KRT81 at ≈27 kDa, corresponding to the size of a protein product translated from a *tKRT81* RNA transcript. HSP90 was used as a loading control. D) An example gene coverage plot from a high *tKRT81* expressing TCGA‐BRCA sample (red) compared to a low *tKRT81* expressing patient library (blue). E) A bar graph (left) and pie charts (right) showing the binning of stratified TCGA‐BRCA patient samples (*n* = 531) based on the PAM50 subtype. *p < 0.05, significance calculated by chi‐square test. F) High *tKRT81* expression in the TCGA‐BRCA dataset has a statistically significant inverse correlation with overall survival as calculated by the log‐rank test. G) Transcript coverage plot of KRT81 gene coverage of a small number of samples from the TCGA‐PAAD dataset.

Interestingly, alignment of the RNA‐sequencing reads to the genome showed that only the last 5 out of 9 exons were ectopically expressed in the lung‐metastatic CTC derivatives (LuM1, LuM2) and the parental line BRx07, with the lung‐metastatic CTC derivatives having much higher expression compared to the parental CTCs (**Figure** **2**A).

This same expression pattern was observed in some commonly used breast cancer cell lines (MCF7, MDA‐MB‐231, MDA‐MB‐361), whereas full‐length *KRT81* was normally expressed in skin cells (Figure 2A). A previous study identified a methylation‐responsive cryptic promoter containing 2 proximal Sp1 binding sites within the fourth intron of the *KRT81* gene that was involved in generating the truncated isoform.^[^ ^20^ ^]^ While the full‐length mRNA encodes for an intact keratin including the N‐terminal keratin head domain, the conserved central rod domain, and the C‐terminal tail domain, the truncated isoform expressed by exons 5–9 encodes for only the C‐terminal half of the rod and tail domains (Figure 2B). Expression of *tKRT81* was confirmed by western blot in the LUM1 and LUM2 cells but not the parental BRx07 cells, showing a ≈27 kDa band corresponding to the smaller protein product translated from the truncated transcript (Figure 2C).

To determine the relevance of *tKRT81* expression within a larger patient dataset, RNA sequencing data available from breast cancer patients in TCGA (TCGA‐BRCA) were analyzed and 531 patient samples that express *tKRT81* were identified. These libraries were stratified into high, medium, and low quantiles based on the gene expression levels of *tKRT81* (Figure 2D). Interestingly, although nearly half of all the libraries were comprised of the luminal subtype, the basal subtype was significantly over‐represented in the high *tKRT81* expression group (*p* < 2.2 × 10^−16^ based on chi‐square test) (Figure 2E), and high *tKRT81* expression was significantly correlated with decreased survival probability (Figure 2F).

In addition to breast cancer, KRT81 has also been examined in other cancer types including non‐Hodgkin's lymphoma,^[^ ^14^, ^17^ ^]^ gastric cancer,^[^ ^15^, ^18^ ^]^ melanoma,^[^ ^19^ ^]^ and pancreatic ductal adenocarcinoma (PDAC).^[^ ^16^ ^]^ In PDAC, KRT81 is being investigated as a marker for an aggressive subtype.^[^ ^16^ ^]^ However, many of the primers, probes, and antibodies used in these studies cannot differentiate between the full‐length and truncated isoforms. We, therefore, compared the RNA sequencing read alignments to the KRT81 gene in the TCGA pancreatic adenocarcinoma (TCGA‐PAAD) dataset and found evidence of *tKRT81* expression in at least 8 patient samples, indicating a need for further clarification regarding the isoforms of KRT81 and their clinical significance in various cancer types (Figure 2G).

### 2.3 *tKRT81* Physically Interacts with *KRT18* and Disrupts Intermediate Filament Organization and Desmosome Structure

**Figure 3:**
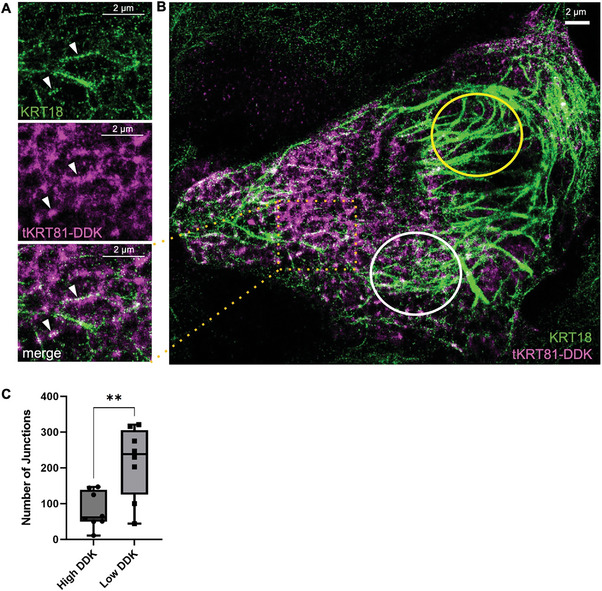
*tKRT81* physically interacts with *KRT18* and disrupts intermediate filament organization. A,B) Representative super‐resolution imaging pictures with antibodies against *KRT18* (green) and DDK (magenta, *tKRT81*) in MDA‐MB‐361 cells that were ectopically transfected with a plasmid expressing *tKRT81*‐DDK. A) Zoomed‐in picture of the area with detectable *tKRT81*. White arrow heads showing the co‐localization of *tKRT81* with *KRT18*. B) Areas of high *tKRT81* expression (white circle) have *KRT18* filaments with diminished integrity, whereas areas with low *tKRT81* expression (yellow circle) show thick *KRT18* filament structures. C) Quantification of *KRT18* filament integrity in high and low *tKRT81* expression areas measured by the mean density of junctions using the Skeleton plugin in Fiji. Mean ± SEM, *n* = 8 ROI per condition. ****p < 0.0001, statistical significance was calculated by unpaired *t*‐test.

Whereas keratin assembly normally occurs in the presence of full‐length keratins, we hypothesized that the expression of *tKRT81*, a type II keratin, will interact with type I keratins at some point in the assembly process to impact the structure and function of keratin IFs in breast cancer cells. To determine *tKRT81* binding factors, we conducted mass spectrometry analysis using MDA‐MB‐361 cells that express a relatively high level of *tKRT81*. We used CRISPR‐mediated interference with sgRNA against the cryptic promotor of *tKRT81* to knockdown *tKRT81* (sg10) and then ectopically expressed *tKRT81*‐DDK‐MYC via lentiviral induction (Figure S1A, Supporting Information). After using anti‐MYC beads to immunoprecipitate interacting proteins, mass spectrometry analysis identified *KRT18*, a type I keratin, as an interacting factor (Figure S1B, Supporting Information). Corroborating our finding, a search in a human binary interactome atlas created through a high‐throughput two‐yeast hybrid system also identified a physical association between the two proteins.^[^ ^21^, ^22^ ^]^ The physical interaction between *tKRT81* and *KRT18* was further verified by colocalization in STORM super‐resolution microscopy^[^ ^23^, ^24^ ^]^ of MDA‐MB‐361 sg10 cells transiently transfected with the *tKRT81*‐DDK‐MYC plasmid (**Figure** **3**). Microscopy showed *tKRT81*‐DDK‐MYC signal localizing to filamentous *KRT18* and forming brush‐like hubs surrounding the *KRT18* filaments (Figure 3A). Interestingly, areas of the cell that displayed high *tKRT81* expression showed diminished integrity of *KRT18* filaments (white circle Figure 3B). In contrast, areas that showed low *tKRT81* expression showed thicker, more intact *KRT18* filaments (yellow circle Figure 3B). To quantify the differences in *KRT18* integrity, we utilized the Skeleton plug‐in feature in Fiji^[^ ^25^, ^26^ ^]^ (Figure S2B–E, Supporting Information) to measure the number of junctions in the *KRT18* bundles in regions with either high or low *tKRT81*‐DDK‐MYC expression, in which a higher number of junctions is correlated with more intact *KRT18*.^[^ ^26^ ^]^ Regions with high *tKRT81*‐DDK‐MYC expression had significantly less junctions and branches than the low *tKRT81*‐DDK‐MYC expression regions (Figure 3C; Figure S2E, Supporting Information) in line with the more diffuse *KRT18* bundles observed in the high *tKRT81*‐DDK‐MYC regions (Figure S2C,D, Supporting Information). Notably, this pattern of *KRT18* filament disruption was not observed when cells were transiently transfected with a full‐length KRT81‐DDK‐MYC plasmid (Figure S3A, Supporting Information), and skeletonized quantification of these full‐length KRT81‐DDK‐MYC expressing regions yielded significantly more junctions indicating higher *KRT18* integrity compared to cells expressing the truncated isotype (Figure S3B,C, Supporting Information).

**Figure 4:**
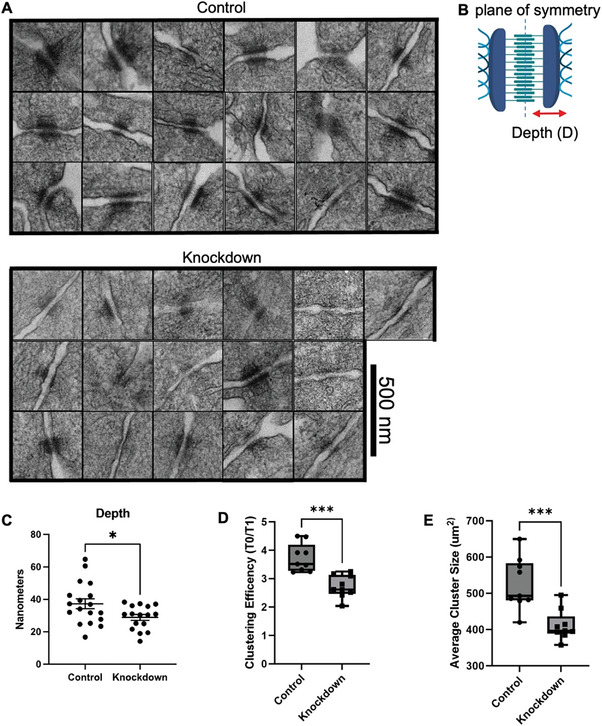
Truncated KRT81 expression is associated with greater desmosomal depth and cluster formation in MDA‐MB‐361 cells. A) Transmission electron microscopy (TEM) images of desmosomes from control and knockdown cells. Scale bar = 500nm for all images. B) A schematic depicting the desmosome plaque depth being measured. C) Quantification of the desmosomal plaque depths between the control and knockdown conditions. *p = 0.0282, Statistical significance was calculated by unpaired *t*‐test. D) Quantification of the clustering efficiency in *tKRT81* expressing control or knockdown cells after 1 h compared to time 0. Mean ± SEM, *n* = 9. ***p < 0.0002, Statistical significance was calculated by unpaired *t*‐test. E) Quantification of the average cluster size in *tKRT81* expressing control or knockdown cells after 1 h. Mean ± SEM, *n* = 9. ***p < 0.0002, Statistical significance was calculated by unpaired *t*‐test

There were also observable differences in desmosomal structures between *tKRT81* expressing control and knockdown cells when imaged by transmission electron microscopy (**Figure** **4**A). In the *tKRT81* expressing control cells, the desmosomes more frequently appeared to be electron‐dense and to form thicker mirror image plaques across the extracellular space of 2 adjacent cells. When *tKRT81* was knocked down, we observed that desmosomes formed thinner electron‐dense plaques close to the cell membrane. To quantify this observation, the desmosome depths (Figure 4B) were measured in a blinded study using randomized images. (Figure 4D). Results showed the depths of desmosomes were significantly thinner in *tKRT81* knockdown cells compared to the controls (Figure 4C), suggesting changes to the inner dense plaque region of the desmosomes where keratin filaments attach to create the scaffold that mediates contact between adjacent cells.^[^ ^27^, ^28^ ^]^

Given that the major functions of desmosomes are related to cell‐cell adhesion, we examined the effects of *tKRT81* expression on cell clustering using a microfluidic device known as the TetherChip.^[^ ^29^, ^30^ ^]^ Knockdown of *tKRT81* in MDA‐MB‐361 cells significantly reduced cell clustering efficiency compared to the *tKRT81* expressing control cells (Figure 4D), as well as the average size of clusters observed (Figure 4E; Figure S4, Supporting Information). Together, this indicates that *tKRT81* expression alters desmosome structure and increases cluster formation in breast cancer cells.

### 2.4 *tKRT81* Alters Cell Stiffness and Morphology

**Figure 5:**
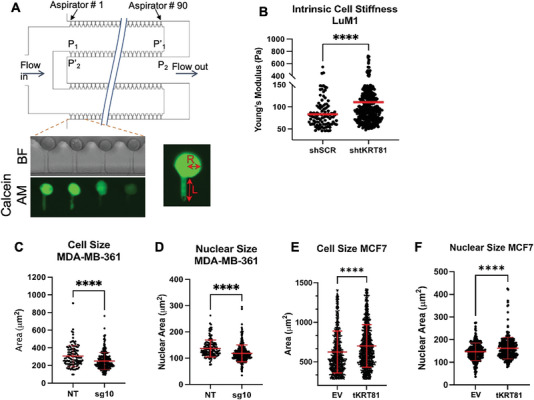
Expression of *tKRT81* alters cell stiffness and morphology. A) Schematic of the high‐throughput microfluidic aspiration (MFA) device. Cells are labeled with live cell dye Calcein AM and then applied to the device, which can assay 1440 cells simultaneously. For each cell inside a channel, the aspiration length (*L*) and cell radius (*R*) are measured and used to determine different cell stiffness parameters. B) Dot plot graph showing the Young's Modulus measurement of individual cell stiffness using the MFA device in LuM1 control (shSCR) and *tKRT81* knockdown (sh*tKRT81*) cells. Red bar = median with 95% confidence interval. ****p < 0.0001, Statistical significance was calculated by Mann–Whitney *t*‐test. C,D) Distribution of cell size measured based on the F‐actin staining (C) and nuclear size measured based on DAPI staining (D) in MDA‐MB‐361 control (NT) and *tKRT81* knockdown (sg10) cells. ****p < 0.0001, Statistical significance was calculated by Mann–Whitney *t*‐test. E,F) Distribution of cell size measured based on the F‐actin staining (C) and nuclear size measured based on DAPI staining (D) in MCF7 control (EV) and *tKRT81* overexpressing (tKR81) cells. ****p < 0.0001, Statistical significance was calculated by Mann–Whitney *t*‐test.

We next evaluated the potential biophysical changes induced by *tKRT81* expression. To examine cell stiffness, a high throughput microfluidic pipette aspiration (MPA) assay was performed. This experiment utilizes a high throughput microfluidic device designed to hold 1440 channels distributed over 16 rows. Each of the channels can trap a single cell in order to produce a stiffness measurement. Cells are flowed onto the chip, and a vacuum is applied in order to trap a cell in the channel. The applied negative pressure of the vacuum draws a portion of the cell membrane into a fixed‐size channel, where a fluorescently labeled cell (Calcein‐AM) can be visualized to measure the radius of the cell (*R*) and the length of the cell deformation in the channel (*L*) (**Figure** **5**A). These measurements are used to calculate the Young's Modulus of each cell within a channel. LuM1 cells that express *tKRT81* were used to generate isogenic knockdown cell lines using shRNA against *tKRT81* (Figure S5A, Supporting Information). Knocking down *tKRT81* (sh*tKRT81*) led to a statistically significant increase in intrinsic cell stiffness (Figure 5B) compared to the *tKRT81*‐expressing controls (shSCR). In addition to increases in cell stiffness, *tKRT81* knockdown in MDA‐MB‐361 cells led to significant decreases in median cell size, as measured by the area of F‐actin signal (Figure 5C) and median nuclear size, measured by the area of DAPI signal (Figure 5D). We also examined changes in cell and nuclear size in MCF7 control and *tKRT81* overexpression cell lines and found a similar trend where overexpression of *tKRT81* resulted in larger cell size (Figure 5E) and nuclear size (Figure 5F).

### 2.5 *tKRT81* Enhances Cell Adhesion to Collagen and Transendothelial Migration

**Figure 6:**
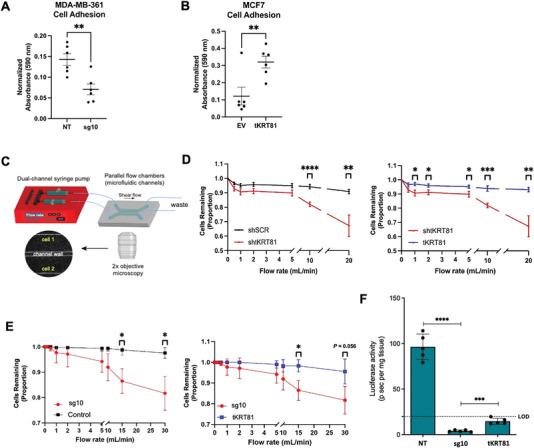
*tKRT81* promotes adhesion to collagen and transendothelial migration. A,B) Graphs showing cell adhesion assay in MDA‐B‐361 control (NT) and *tKRT81* knockdown (sg10) (A) and MCF7 control (EV) and *tKRT81* overexpression (*tKRT81*) cells (B). C) Schematic diagram of a dual‐channel microfluidic device to assay cell adhesion under shear stress conditions. D,E) Graphs showing the percentage of cells that remain adhered to collagen‐coated channels after application of shear force with increasing flow rates in LUM1 control (shSCR) and *tKRT81* knockdown cells (sh*tKRT81*) (D) and MDA‐MB‐361 control (NT), knockdown (sg10), and rescue cells (E). The left graph for each respective cell line shows *tKRT81* control and knockdown (sg10) cells and the right graph shows *tKRT81* knockdown (sg10) and *tKRT81* overexpression (*tKRT81*) cells. *n* = 3, *p < 0.05, **p < 0.01, ***p < 0.005, and ****p < 0.0001. Significance was calculated with an unpaired *t*‐test in each condition. F) Graph showing the number of transmigrated control (NT), tKR81 knockdown (sg10), or *tKRT81* overexpression (*tKRT81*) MBA‐MD‐361 cells in mouse lung. Ex vivo luciferase activity was quantified from lysates of whole mouse lung extracted 24 h after intracardiac injection. *n* = 5, ***p < 0.005 and ****p < 0.0001. Significance was calculated by a two‐tailed unpaired *t*‐test, mean ± SEM.

To examine the effect of *tKRT81* on cell adhesion, we performed cell adhesion assays on collagen‐coated plates. Knockdown of *tKRT81* in MDA‐MB‐361 cells (sg10) significantly reduced adhesion compared to the control (NT) (**Figure** **6**A), whereas in MCF7 cells, overexpressing *tKRT81* increased adhesion compared to the control (EV) (Figure 6B). Similarly, we tested adhesion under shear stress conditions using a microfluidic chip attached to a dual‐channel syringe pump (Figure 6C). The microfluidic chip is coated with collagen I, and cells are added to the channels and allowed to adhere. The number of cells that have adhered is quantified as “before shear flow”. Increasing amounts of shear flow are then applied (ramping up from 0 to 30 mL min^−1^, with 10 s holding of each flow rate in a stepwise fashion)^[^ ^31^ ^]^ and the number of cells left attached to the surface is quantified at each rate of flow and quantified as “after shear flow”. Adhesion to collagen under shear stress conditions was significantly greater in *tKRT81* expressing LuM1 control cells (shSCR) and MDA‐MB‐361 control cells (NT) compared to isogenic LUM1 *tKRT81* knockdown cells (sh*tKRT81*) or MDA‐MB‐361 knockdown cells (sg10), and this phenotype could be rescued by *tKRT81* overexpression (*tKRT81*) in the same cell lines (Figure 6D,E). Notably, this significant difference in adhesion under shear‐stress was only observed on surfaces coated with collagen. There were no observable differences in adhesion under shear stress conditions on surfaces coated with membrane‐bound ICAM1 (*mb*‐ICAM‐1), immobilized ICAM1 (*im*‐ICAM‐1), and fibronectin (Figure S5B–D, Supporting Information). Since cytoplasmic keratins have been reported to localize proteins to either the cell surface or cytoplasm,^[^ ^4^, ^5^, ^32^ ^]^ we tested whether the collagen binding protein integrin β1 localization was affected by *tKRT81* expression to determine if this was the mechanism behind the observed adhesion phenotype. However, flow cytometry analysis in LuM1 and MDA‐MB‐361 control, *tKRT81* knockdown, and rescue cells showed no difference in cell surface expression of integrin β1 (Figure S5E,F, Supporting Information). In addition to adhesion phenotypes, keratins have also been implicated in migratory and invasive cell behaviors.^[^ ^33^ ^]^ Although there was no difference observed with in vitro transwell migration (Figure S5G, Supporting Information) or invasion (Figure S5H, Supporting Information), a significant decrease with in vivo lung transendothelial migration was observed in MDA‐MB‐361 sg10 cells lacking *tKRT81* using a highly sensitive luciferase activity assay on whole lung lysates harvested 24 h after tail vein injection (Figure 6F). Although the *tKRT81* rescue cells (*tKRT81*) had significantly higher luciferase activity compared to *tKRT81* knockdown cells (sg10), a full rescue of the strong metastatic phenotype observed in the control cells (NT) was not observed (Figure S5I, Supporting Information). To examine why this may be the case, we visualized the *tKRT81*‐DDK‐MYC overexpression in the rescue cells using immunofluorescence microscopy against the DDK tag and found that the overexpression of DDK is highly heterogeneous (Figure S5J, Supporting Information). Given that lungs are harvested only 24 h after tail vein injection for this assay, heterogeneity of overexpression in the *tKRT81* rescue cells may result in a lower number of cells entering the lungs in that short time frame compared to the unaltered *tKRT81* expressing control cells.

### 2.6 Expression of *tKRT81* Promotes In Vivo Lung Metastasis

**Figure 7:**
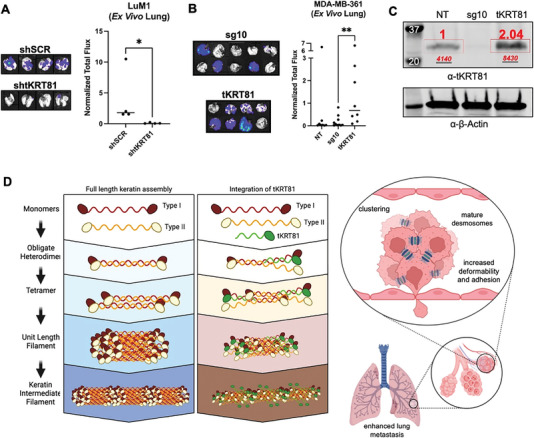
*tKRT81* upregulation promotes lung metastasis in vivo. A) Ex vivo bioluminescent lung imaging (left) and quantification (right) of mice 32 weeks after tail vein injection of LuM1 control (shSCR) and *tKRT81* knockdown (sh*tKRT81*) cells. *p = 0.0143, significance is calculated by one‐tailed Mann–Whitney *t*‐test. B) Ex vivo bioluminescent lung imaging (left) and quantification (right) of mice 11 weeks after tail vein injection of MDA‐MB‐361 control (NT) and *tKRT81* knockdown (sg10) and overexpression rescue (*tKRT81*) cells. **p = 0.0031, significance is calculated by one‐tailed Mann–Whitney *t*‐test. C) Immunoblotting with antibodies against KRT81 and β‐Actin for cells used in (B). Red numbers were the normalized quantification of *tKRT81*. D) Schematic representation of *tKRT81* promotion of lung metastasis in breast cancer cells.

We then evaluated the effect of *tKRT81* expression on tumorigenesis and metastasis in immunodeficient NSG mice. Primary tumors generated by mammary fat pad orthotopic injection of 2.5 × 10^5^ LuM1 cells with *tKRT81* expression control (shSCR) or knocked down (sh*tKRT81*) showed no significant difference in size and growth over the period of 26 weeks (Figure S6A, Supporting Information). To test for lung recolonization ability, the same cells were injected by lateral tail vein, and mice were monitored by bioluminescent imaging for a period of 32 weeks. At the experimental endpoint, although there was no statistical significance in whole body bioluminescent signal (Figure S6B, Supporting Information), there was a significant difference in lung tumor burden when imaged ex vivo (**Figure** **7**A). Similar lateral tail vein injections with the MDA‐MB‐361 *tKRT81* expressing control (NT), *tKRT81* knockdown (sg10), and rescue (*tKRT81*) cell lines showed a significant difference between the rescue and knockdown groups both by whole‐body imaging (Figure S6C, Supporting Information) and in ex vivo measured lung tumor burden (Figure 7B). Although we did not detect any differences between the *tKRT81*‐expressing control and *tKRT81* knockdown groups, this may be due to differences in total protein expression levels. The rescue cell line has a twofold higher abundance of *tKRT81* than the endogenous levels present in the control cell line (Figure 7C), and therefore, an in vivo phenotype may become more evident if the experiment is carried out for a longer period of time.

## 3 Discussion

In this study using immune‐deficient mouse models, we found that *tKRT81* was upregulated in the in vivo lung metastases generated from patient‐derived CTC lines. Upregulation in the primary tumor was significantly correlated with lung metastasis‐free survival in a small cohort of breast cancer patients and overall survival in the large TCGA‐BRCA datasets. Expression of *tKRT81* was associated with the disruption of the keratin filament network in the cytoplasm and with more electron‐dense desmosomes. Desmosome depth was higher in *tKRT81* expressing cells compared to knockdown cells, suggesting changes to the inner dense plaque region of the desmosome structure in which keratin filaments attach to desmosome proteins.^[^ ^27^, ^28^ ^]^ Our discovery that desmosomes are altered by *tKRT81* expression in cancer cells is a novel finding of a truncated keratin filament influencing desmosome structure and cell‐cell adhesions, resulting in increased cell clustering, which has significant implications in CTC biology and metastasis. Furthermore, *tKRT81* was discovered to physically interact with and disrupt the filamentous organization of *KRT18* in breast cancer cells. The cytoskeletal changes associated with the expression of *tKRT81* resulted in larger cells that had larger nuclei, were softer, and were more elastically deformable, potentially contributing to cellular capability for extravasation within the metastatic cascade. Knocking down *tKRT81* decreased cell adhesion to collagen with and without the application of shear stress, and these *tKRT81* knockdown cells also demonstrated significantly less transendothelial migration to the lung in vivo compared to *tKRT81* expressing control cells. The lack of significant difference observed in the invasion and migration potential in vitro indicates the involvement of additional factors unique to the in vivo environment. One possibility involves the increased clustering of *tKRT81* cells, as CTC clusters have been shown to increase the rates of survival and entrapment in the small capillaries of distal organs like the lung,^[^ ^34^ ^]^ aspects which are far less relevant in vitro. Further, in vivo experiments also show increased lung metastasis due to *tKRT81* expression, which can be abrogated by knocking down *tKRT81* levels and provides additional evidence for the pro‐metastatic role of *tKRT81* in vivo. These data point to a working model in which upregulation of *tKRT81* leads to alterations in the cell cytoskeleton that increase cell deformability, adhesion, and cluster formation through greater desmosome depth, leading to enhanced lung metastasis (Figure 7D).

In the literature, KRT81 appears in numerous cancer studies. A single nucleotide polymorphism in the 3′UTR of the KRT81 gene is associated with a higher risk of gastric cancer,^[^ ^15^ ^]^ worse prognosis in non‐Hodgkin's lymphoma,^[^ ^14^ ^]^ increased recurrence in non‐small‐cell lung cancer,^[^ ^17^ ^]^ and lower survival in multiple myeloma.^[^ ^18^ ^]^ KRT81 is also currently being developed as a marker for quasi‐mesenchymal pancreatic ductal adenocarcinoma, the most aggressive subtype of pancreatic cancer,^[^ ^16^ ^]^ but whether the full‐length or truncated isoform is expressed should be further investigated. KRT81 was identified in several breast cancer studies as well. It was identified, but not further explored, in a study that generated mouse models of lung metastasis using the MDA‐MB‐231 breast cancer cell line to develop a 54‐gene panel comprising a breast cancer lung‐metastasis gene signature.^[^ ^35^ ^]^ Another group looking at genes involved in GATA3‐mediated lung metastasis found that GATA3 can repress KRT81 and several other genes involved in breast‐to‐lung metastasis.^[^ ^36^ ^]^ Gene expression profiling between pure breast ductal carcinoma in situ (DCIS) cells with DCIS associated with synchronous invasive breast cancer identified KRT81 upregulation as 1 of 9 genes associated with synchronous invasive breast cancer.^[^ ^37^ ^]^ In the most recent study, one group found that both full‐length and truncated KRT81 could be detected in both normal and breast cancer cell lines and that knockdown resulted in diminished in vitro migration and invasion of the MDA‐MB‐231 cell line.^[^ ^38^ ^]^ Another study performed single‐cell RNA sequencing of the “normal” adjacent tissue of a patient diagnosed with DCIS using the 10X Genomics platform. Sequencing analysis identified KRT81 expression in 1 of 3 clusters of epithelial cells. The identity of this KRT81‐expressing cluster was ambiguous, however, due to the expression of both luminal and basal epithelial keratins, as well as an overall signature most similar to triple‐negative breast tumors. Moreover, it is unclear whether the expression was of the full length or truncated KRT81 due to the inherent 3′bias of the 10X Genomics platform.^[^ ^39^ ^]^ Our study is the first to demonstrate an association between high *tKRT81* expression in patient‐derived CTCs metastasized to the lung in vivo, as well as an association between high *tKRT81* and poor survival in breast cancer patients. We also demonstrate the functional changes induced by the truncated form of KRT81. The data presented highlights the need to further investigate whether the full‐length or truncated isoforms are associated with more aggressive cancer‐type characteristics. While there have been many correlational studies implicating KRT81 in breast cancer and other types of aggressive cancers, many of the probes and primers used in previous studies are unable to distinguish between the full‐length and truncated forms, contributing to the lack of clarity in the functional implications of *tKRT81* expression. Our study provides novel insights into the role of *tKRT81* in modifying the mechanical properties of the tumor cells to promote breast cancer metastasis.

Our data showed that *tKRT81* expression fine‐tunes modulation of the physical properties of the cell to promote metastasis. Although further studies are needed, it is conceivable that softer, more deformable primary tumor cells that are already molecularly primed for uninhibited proliferation can resist the physical stressors of the circulatory environment that encourage cell rupture and destruction and are physically selected for completion of the metastatic cascade, in addition to the mentioned survival benefits of increased clustering efficiency.

These results also provide a compelling argument for improved mapping of the keratin landscape in various cancers. Keratins comprise the largest family of IF proteins by far, yet are relatively understudied and not as well characterized as other cytoskeletal components beyond their frequent use in tumor diagnostics. A more detailed understanding of the regulation, structure, and function resulting from the combinatorial expression of various keratins, particularly potential other truncated variants like *tKRT81*, in different types of cancers may be useful for the quantification of metastatic propensity, as well as prognostic implications for patients.

## 4 Experimental Section

### Cell Culture

BRx07 CTCs and the corresponding isogenic, in vivo‐selected lung‐metastatic derivatives, LuM1 and LuM2, were isolated and cultured as previously described.^[^ ^13^, ^40^ ^]^ Briefly, these cells were cultured in suspension on ultra‐low attachment tissue culture plates in 4% O_2_ and 5% CO_2_ in CTC media (RPMI 1640 medium, 20 ng mL^−1^ EGF, 20 ng mL^−1^ bFGF, 1X B27, and 1X antibiotic/antimycotic). The adherent cell lines used in this study were purchased from American Type Culture Collection (ATCC) and cultured in DMEM, 10% FBS, 1% Penicillin‐Streptomycin (MDA‐MB‐231, MDA‐MB‐361, and MCF7) or RPMI, 10% FBS, 1% Penicillin‐Streptomycin (T47D). All cell lines were routinely tested for mycoplasma contamination using a commercially available kit (MycoAlert, Lonza Group).

### Construct Generation

MYC‐DDK‐tagged truncated KRT81 construct was cloned from Lenti ORF clone of Human keratin 81, myc‐ddk‐tagged, from Origene (Catalog# RC220726L3). This construct was modified by PCR cloning to move the puromycin resistance gene upstream of the P2A sequence followed by the truncated KRT81 sequence that is C‐terminally tagged with MYC and DDK before the stop codon.

The shRNA sequence targeting human KRT81 used was: (sh1) 5′‐CCG GCA CTC CTG GCC TCA CAT TTC TCT CGA GAG AAA TGT GAG GCC AGG AGT GTT TTT TG‐3′ and (sh2) 5′‐CCG GAG CAA GTG CTC AGC TAC TTC TCT CGA GAG AAG TAG CTG AGC ACT TGC TTT TTT TG‐3′ (both targeting the 3′UTR) and was cloned into pLKO.1 Tet‐on neo vector. Unless labeled, the sh*tKRT81* refers to cells generated using sh1. Similarly, the shRNA for the non‐targeting scramble (SCR) control sequence used was: 5′‐CCG GCC TAA GGT TAA GTC GCC CTC GCT CGA GCG AGG GCG ACT TAA CCT TAG GTT TTT G‐3′. Doxycycline was used at a final concentration of 100 ng mL^−1^ for 48 h to induce hairpin constructs.

The two‐part CRISPR inactivation lentiviral system was purchased from Addgene: Lenti‐dCas9‐KRAB‐Blast (Addgene, 89 567) and pLKO5.sgRNA.EFS.tRFP657 (Addgene, 57 824). The online Gene Perturbation Platform (GPP) sgRNA Designer from the Broad Institute was used to design sgRNAs (sg10) targeting intron 4 of the KRT81 gene (5′‐ CAC CGA AAC CTG GCA GCC AGC AGA G) and cloned into the pLKO5.sgRNA.EFS.tRFP657 vector as directed in the protocol associated with the product. Cells were first transduced with the dCas9‐Krab lentivirus, selected by blasticidin concentrations optimized for the different cell lines, then subsequently transduced with the sgRNA containing lentivirus and purified for RFP657 positive populations by FACS.

### Lentiviral Production and Generation of Stable Cell Lines

Lentivirus was generated using the published protocol from The RNAi Consortium (TRC) Broad Institute. In brief, low passage 293T cells were co‐transfected with second‐generation lentiviral packaging vectors and the aforementioned expression constructs using TransIT‐LT1 transfection reagent (Mirus). The next day, cells were cultured in a high serum growth medium and collected at both 48 and 72 h post‐transfection. Viral media was either used directly to transduce adherent cell lines or concentrated with a Lenti‐X concentrator (Clontech) and resuspended in PBS to remove serum for transduction of CTCs and lung‐metastatic CTC derivatives. All cells were transduced with the virus in the presence of 8 µg mL^−1^ polybrene and selected by 1‐week antibiotic selection based on experimentally derived kill curve assays, or by FACS.

### Western Blot

Cells were washed in PBS and lysed in Laemmli Buffer (50 mm Tris pH 6.8, 1.25% SDS, 15% glycerol) and heated at 95 °C for 15 min. After protein quantification by Lowry protein assay (Bio‐Rad) samples were reduced with 5% (v/v) beta‐mercaptoethanol. Lysates were then mixed with bromophenol blue (0.01% v/v) and run on a denaturing 4–15% Mini‐PROTEAN TGX Precast gels (Bio‐Rad). Gels were transferred by semi‐dry method to low fluorescence PVDF membranes in a Trans‐Blot Turbo Transfer System (Bio‐Rad) using the standard Bio‐Rad listed protocol. Membranes were then blocked in 5% NFDM in TBS followed by primary antibody incubation in blocking buffer with 0.2% Tween 20 at 4 °C overnight. Membranes were then washed (3 × 10 min each, 1XTBST) and incubated with LICOR secondary antibodies diluted in blocking buffer with 0.2% Tween 20 and 0.01% SDS for 1 h at RT. Membranes were then washed (3 × 10 min each, 1XTBST), rinsed in TBS, and imaged on a LI‐COR imaging instrument. The following antibodies were used: Pan‐Keratin (C11) Mouse mAb Cell Signaling 4545S; DYKDDDK Tag (D6W5B) Rabbit mAb Cell Signaling 14793S; Keratin 18 (DC10) Mouse mAb Cell Signaling 4548; HSP90 antibody Abcam ab13492; Anti‐basic Hair Keratin K81 guinea pig polyclonal, serum Progen GP‐hHb1; KRT81 polyclonal antibody ProteinTech 11342‐1‐AP; APC Anti‐integrin beta 1 antibody [P5D2] Abcam ab221241; b‐Actin (mouse) Sigma A5441; IRDye 800CW goat anti‐rabbit IgG secondary antibody LI‐COR 925–32211; IRDye 680RD goat anti‐mouse IgG secondary antibody LI‐COR 926–68070; IRDye 800CW donkey anti‐guinea pig IgG secondary antibody LI‐COR 926–32411.

### RNA‐seq and Differentially Expressed Genes (DEG) Analysis

RNA was isolated using a Zymo RNA isolation kit. RNA quantity and quality were measured by NanoDrop and TapeStation before the KAPA Stranded RNA‐Seq Kit with RiboErase (HMR) was used to generate the sequencing libraries. Libraries were sequenced at either USC Translational Genomics Core or CHLA. Some libraries were generated and sequenced by Novogene Corporation. CTC sequencing reads were mapped to hg19 (GRCh37) reference using STAR v2.5.2b.^[^ ^41^ ^]^ Genes annotated in the ENSEMBL GRCh37.p13 GTF (release 75) were quantified using HTSeq‐count.^[^ ^42^ ^]^ Differential analysis was performed using DESeq2.^[^ ^43^ ^]^ Differential genes (DE) across the target metastatic sites (FDR ≤ 0.05) were identified after controlling for the cell line, dissociation, and culture effects. Coverage tracks were created using deepTools^[^ ^44^ ^]^ for visualization in the UCSC genome browser. RNA‐seq data of different breast cancer cell lines were obtained from Daemen et al. 2013.

### Clinical Data Analysis

The GSE12276 dataset was analyzed in Partek Genomics Suite 6.6. Kaplan–Meier survival curves were calculated on *KRT81* expression split into two quantiles based on the median value. Comparisons were considered statistically significant by log‐rank *p*‐value < 0.05.

TCGA‐BRCA data were analyzed. All TCGA‐BRCA data were downloaded and RNA sequencing reads aligned to exons 1–9 of *KRT81*. Libraries expressed *tKRT81* were selected for survival analysis. Patients across different subtypes with high *tKRT81* expression levels (67th percentile) were significantly associated with decreased overall survival compared to those patients with low expression. The RNA‐seq BAM files of TCGA‐BRCA patients were downloaded using the GDC data transfer tool. For each tumor library, reads falling into exons 1–9 were quantified and counts were normalized for the library size. To identify expression levels of full‐length KRT81 and *tKRT81* transcripts, a goodness of fit test is performed based on read counts in exons 1–4 and exons 5–9. The coverage at base pair resolution across the gene body is calculated and the coverage plots were also compared to identify *tKRT81* expression. Only libraries with TPM expression ≥5 were considered for the analysis. An FDR level of 0.1 is used for multiple testing corrections. Gene body coverage for house‐keeping genes was calculated using RSeQC^[^ ^41^ ^]^ and libraries that showed 3′ coverage bias were filtered out. Libraries were stratified into high or low *tKRT81* expression and clinical data of the patients were obtained using TCGAbiolinks R package.^[^ ^42^ ^]^ Survival analyses were performed using the survival R package.^[^ ^45^ ^]^

### Immunocytochemistry

Cells were seeded on 18 mm Coverglass No. 1 coverslips and cultured overnight. After washing with PBS, cells were fixed with 4% paraformaldehyde in PBS for 10 min at RT, washed in PBS, then permeabilized with 0.1% Triton X‐100 in PBS for 10 min at RT. Cells were then blocked for 1 h at RT in 5% goat serum in PBS with 0.1% Tween 20. Primary antibodies were diluted in blocking buffer and incubated with coverslips overnight at 4 °C. After 3 × 5 min washes with PBST, secondary antibodies were diluted in blocking buffer and incubated with coverslips for 1 h at RT in the dark. The coverslips were then washed for 3 × 5 min in PBST, stained with a nuclear dye 4,6‐dianidino‐2‐phenylindole (DAPI) for 5 min, and mounted using ProLong Gold Antifade Mounting media (Thermo Fisher) overnight in the dark. The next day, coverslips were imaged using the Keyence BZ‐X810 microscope. Cell parameters were quantified using the Hybrid Cell Count and Macro Cell Count features of the BZ‐X800 Analyzer Software.

### Super‐Resolution Microscopy and Quantification

3D‐STORM super‐resolution microscopy was performed as described previously.^[^ ^23^, ^24^, ^45^ ^]^ In brief, cells were seeded on 18 mm Coverglass No. 1 coverslips and cultured overnight. After washing with PBS, cells were fixed with 4% paraformaldehyde in PBS for 10 min at RT, washed in PBS, and permeabilized and blocked in blocking buffer (3% w/v BSA, 0.1% v/v Triton X‐100 in PBS) for 1 h. Afterward, the cells were incubated with primary antibodies (below) in the blocking buffer for 12 h at 4 °C. After washing in a washing buffer (0.3% w/v BSA and 0.01% v/v Triton X‐100 in PBS) three times, the cells were incubated with dye‐labeled secondary antibodies (below) for 1 h at room temperature. Then, the samples were washed three times with the washing buffer and three times with PBS. Primary antibodies used: DYKDDDDK Tag (D6W5B) (Cell Signaling Technologies 14793S) and *KRT18* (DC10) (Cell Signaling Technologies 4548S). Secondary antibodies used: Alexa Fluor 647‐labeled goat anti‐mouse (Invitrogen A21240) and CF‐568 conjugated to AffiniPure donkey anti‐rabbit IgG (H+L) (Jackson ImmunoResearch 711‐005‐152). 3D‐STORM super‐resolution microscopy^[^ ^23^, ^24^ ^]^ was carried out on a homebuilt setup using a Nikon CFI Plan Apo λ 100x oil immersion objective (NA 1.45).^[^ ^46^ ^]^ The sample was mounted with an imaging buffer consisting of 5% (w/v) glucose, 100 mm cysteamine, 0.8 mg mL^−1^ glucose oxidase, and 40 µg mL^−1^ catalase in a Tris‐HCl buffer (pH 7.5). For two‐color imaging, the two targets were labeled by Alexa Fluor 647 and CF568, respectively, and were imaged sequentially using 647 and 560 nm excitation lasers. These lasers were passed through an acousto‐optic tunable filter and illuminated a few micrometers into the sample at ≈2 kW cm^−2^, thus photoswitching most of the labeled dye molecules in the sample into the dark state while allowing a small, random fraction of molecules to emit across the wield‐field over different camera frames. Single‐molecule emission was passed through a cylindrical lens of focal length 1 m to introduce astigmatism, and recorded with an Andor iXon Ultra 897 EM‐CCD camera at a framerate of 110 Hz, for a total of ≈50 000 frames per image. The raw STORM data were analyzed according to previously described methods.^[^ ^23^, ^24^ ^]^ Quantification of keratin integrity from STORM images was performed using the Analyze Skeleton plugin in Fiji, described previously.^[^ ^25^ ^]^ Briefly, the *KRT18* channel images were converted to a binary image with a constant threshold. Regions of interest (ROIs) were selected in high and low DDK areas, and these ROIs were cropped out of the *KRT18* binary image. ROIs were then skeletonized, and the number of junctions and branches was calculated as a metric of keratin bundle integrity.

### Transmission Electron Microscopy

Cells were grown to confluence on collagen‐coated tissue culture plates and then fixed in TEM Buffer (2.5% glutaraldehyde, 2% paraformaldehyde, 7% w/v sucrose in 0.1 m HEPES) and submitted to USC's Core Center of Excellence in Nano Imaging run by senior scientist Dr. Carolyn Marks.

### Immunoprecipitation and Mass Spectrometry

All steps were done on ice and all buffers were supplemented with 1X Roche complete EDTA‐free Protease Inhibitor Cocktail prior to use. Roughly 10^6^–10^7^ cells were seeded in large 15 cm tissue culture plates. The next day, cells were washed in PBS, scraped, and pelleted. Cell pellets were frozen at −80 °C for at least 1 h to help in the lysis process. Thawed pellets were then lysed in 200 µL cold lysis buffer (10 mm Tris‐HCl pH = 7.5, 150 mm NaCl, 0.5 mM EDTA, 0.5% Nonidet P40 Substitute) for 30 min on ice with frequent pipetting. Insoluble debris was pelleted by centrifugation at 17 000 g for 10 min at 4 °C and the resulting supernatant was then diluted to 500 µL in dilution buffer (10 mm Tris‐HCl pH 7.5, 150 mm NaCl, 0.5 mm EDTA). The diluted lysate was precleared using 25 µL of mNeonGreen‐Trap Magnetic Agarose beads (Chromotek ntma). The precleared lysate was then incubated with 25 µL of pre‐equilibrated Myc‐Trap Magnetic Agarose beads (Chromotek ytma) for 1 h at 4 °C or with mNeonGreen‐Trap Magnetic Agarose beads. The beads were then washed 5×5 min each in cold wash buffer (10 mm Tris‐HCl pH 7.5, 150 mm NaCl, 0.5 mm EDTA, 0.05% Nonidet P40 Substitute) and binding protein complexes were eluted in 30 µL of 2X SDS‐Sample buffer (120 mm Tris‐HCl pH 6.8, 20% glycerol, 4% SDS, 0.04% bromophenol blue, 10% beta‐mercaptoethanol). Proteins were then run on 7.5% and 18% SDS‐PAGE gels to resolve high‐ and low‐molecular‐weight proteins, respectively. Gels were then silver stained by sequential incubation in the following buffers made with deionized and distilled water: 50% methanol for 10 min, 5% methanol for 10 min, 32 micromolar DTT solution, 0.1% AgNO_3_ solution for 10 min, two quick rinses with deionized water followed by incubation with developing solution (0.02% paraformaldehyde, 3% Na_2_CO_3_) until bands appear. The developing solution was then neutralized by empirically adding citric acid powder. Differential bands were identified between the control cell line and cell line expressing recombinant *tKRT81*‐DDK‐MYC extracted from the gel and submitted to the mass spectrometry core at USC's School of Pharmacy run by director Dr. Alireza Abdolvahabi. In brief, silver‐stained IP gel pieces were cut and de‐stained using a solution containing 100 mm sodium thiosulfate and 30 mM potassium ferricyanide (1:1 ratio) for 30 min with gentle shaking. Gel pieces were then washed with 25 mm ammonium bicarbonate in 50% acetonitrile (ACN), reduced with 5 mm dithiothreitol (DTT) for 30 min at 60 °C, alkylated with 20 mM iodoacetamide (IAA) at dark for 30 min, and digested overnight with trypsin Gold (Promega) at a final concentration of 6 ng µL^−1^. Digestion was then quenched by the addition of 2% formic acid (FA). Peptides were extracted twice with 50% ACN/2% FA each time for 30 min with vigorous shaking. Extracted peptides were evaporated to complete dryness and reconstituted in 5 µL of MALDI matrix solution (10 mg mL^−1^ dihydroxybenzoic acid in 70% ACN/0.1% FA). Half a microliter of this solution was spotted on a 384 Big Anchor MALDI target, let dry under ambient conditions, and analyzed using a Rapiflex MALDI‐TOF‐TOF mass spectrometer working under Linear Mode. Prior to running samples, the mass spectrometer was calibrated using a peptide calibration solution containing bradykinin, angiotensin, substance P, bombesin, ACTH, and somatostatin. The resulting peptides were searched against the SwissProt library using BioTools software (Bruker Daltonics) for protein identification. The parent mass tolerance was set to 50 ppm. Protein identification was performed using peptide mass fingerprinting (PMF) and validated with MS/MS.

### Cluster Formation Assay by TetherChip

TetherChips (Ju 2020) were generously gifted from Dr. Stuart Martin's lab at the University of Maryland Baltimore. The TetherChip is a microfluidic device with a thermal‐crosslinked polyelectrolyte multilayer nanosurface underlying a lipid layer that enables cell membrane tethering to the optically‐clear microfluidic slides on which the TetherChip nanosurface is plated for spatial immobilization of cells.

To prepare the cells, cells were first trypsinized for less than 10 min and counted to obtain 10 000 cells per well in a single cell suspension. Cells were then seeded either directly onto a TetherChip for a *t* = 0 h timepoint, or into a low‐attachment 96‐well plate. Cells seeded into low attachment 96‐well plates were allowed to cluster for 1 h, before being transferred from suspension into a new TetherChip for a *t* = 1 h timepoint. At each respective timepoints, TetherChips containing cells are incubated at 37 °C for 30 min to fully tether the cells to the lipid layer for immobilization. Cells were subsequently fixed with 4% paraformaldehyde for 10 min at room temperature, followed by co‐staining overnight with DAPI (1:5000) and Wheat Germ Albumin (WGA, 1:500). After overnight incubation, slides are rinsed three times with 1x DPBS. DAPI images were acquired using the Nikon Eclipse Ti‐E inverted microscope at x magnification for quantitative computational analysis. DAPI and WGA images were acquired using the Keyence BZ‐X810 microscope at 10x magnification. For computational analysis of images, DAPI images were loaded into Fiji and binarized using a set threshold of intensity greater than 19. The Analyze Particles Objects application in Fiji was used to quantify clusters. Nuclei smaller than 75 pixels^2^ (a size smaller than a single nucleus) were considered cellular debris and removed from further analysis. Objects larger than 76 pixels^2^ were defined as clusters. Cluster efficiency is defined by comparing the number of individual clusters over time. Values at *t*  =  0 h were divided by the respective final cluster numbers (*t*  =  1 h) for each condition. Average cluster size is defined by the average size of clusters per well at the final experimental timepoint (*t* = 1 h) as determined through the Analyze Particles application. Error bars indicate the standard deviation across experiments. Paired *t*‐test was used to determine statistical significance.

### Microfluidic Pipette Aspiration (MPA) Assay

MPA devices were generated by Shamim Ahmmed at Texas Tech University in Dr. Siva Vanapalli's lab. Microfluidic devices for MPA were made using standard soft lithography.^[^ ^47^ ^]^ The design of MPA contains 1440 aspirator channels with each channel having a cross‐section of 5 mm × 5 mm. One thousand five hundred cells in a 15 µL volume were aspirated using a vacuum pump (Fluigent Inc.) at a negative pressure of DP = −600 Pa. Prior to loading of cells in the MPA devices, cells were tagged with Calcein‐AM to enable easy visualization of the aspiration length. Images of trapped cells in the aspirator channels were obtained using a Keyence BZ‐9000 microscope. A custom‐written MATLAB (Mathworks Inc.) routine was developed for processing images and quantifying the equilibrium aspiration length *L*. Young's modulus *E* was calculated using the expression i = 3RDPf/2pL, where *R* is the hydraulic radius of the aspirator channel (= 5 mm) and *f* is the wall function with a typical value of 2.1.^[^ ^48^ ^]^

### Cell Adhesion Assay and Morphology Assessment

Rat tail collagen I was diluted in 0.2 N acetic acid to a final concentration of 100 µg mL^−1^ and used to coat wells for 1 h at RT. The collagen was then gently removed and washed 2x with PBS. The coated ECM was blocked in DMEM with 10% FBS and incubated for 30 min at 37 °C. During this time, cells were trypsinized for 3 min, neutralized, and quickly counted and adjusted to an optimized final seeding density (MDA‐MB‐361: 20 000 cells/well, MCF7: 30 000 cells/well) and adhered to the collagen‐coated wells for 30 min at 37 °C. Unadhered cells were then removed by plate inversion and the wells were washed 2x with cold PBS containing 1 mm CaCl_2_ and 1 mm MgCl_2_. Cells were then fixed with cold methanol for 10 min at RT, followed by three washes with PBS. Crystal violet (0.5% wv crystal violet in 20% ethanol) was incubated on the cells for 10 min at RT with gentle shaking. Excess crystal violet was removed by immersing the plates sequentially in 3 × 2 L beakers of distilled water for 1 min each. Crystal violet was then recovered by adding 200 µL of 100% methanol to each well for 15 min at RT with gentle shaking. Hundred microliters of the recovered crystal violet solution was transferred to flat‐bottom 96‐well plates and absorbance was measured at 590 nm. Adhered cells stained with crystal violet were also imaged at 40X with five random images taken per condition. These images were then randomized and given to three blinded individuals who qualitatively binned cells into two nondescript categories: strongly adhered and weakly adhered. The cell counts were quantified between the three individuals to obtain a quantitative measurement of cell morphology.

### Cell Adhesion Under Shear Stress Conditions

This experiment was performed by Jia Hao from Dr. Keyue Shen's lab at USC, and the following protocol was applied:

*Preparation of Supported Lipid Bilayers and Protein Tethered Surfaces*: Lipid components, 18:1 (Δ9‐Cis) 1,2‐Dioleoyl‐sn‐glycero‐3‐phosphocholine (DOPC) and 5% 18:1 1,2‐dioleoyl‐sn‐glycero‐3‐[(N‐(5‐amino‐1‐carboxypentyl)iminodiacetic acid)succinyl] (nickel salt) (DGS‐NTA(Ni)), dissolved in chloroform were purchased from Avanti Polar Lipids and mixed. The lipids were air‐dried in round‐bottom flasks and desiccated for 2 h with a house vacuum pump in a chemical fume hood. The lipid mixture was resuspended by bath sonication in 1X PBS at a final concentration of 2.5 mg mL^−1^ and extruded 10 times through a membrane with 50 nm pore size (Avanti Polar Lipids) into small unilamellar vesicles (SUVs). The SUV solutions were then diluted 1:1 in 1X PBS (pH 7.4) before being loaded onto the detergent‐cleaned and dried glass coverslip through the loading chamber, and incubated for 2 min to spontaneously form the lipid bilayers. The chambers were then washed with a 10X excess volume of 1X PBS.

*ICAM‐1 Capturing on Lipid Bilayer, Immobilization, and Substrate Coating*: For protein capturing on lipid bilayer, the substrate was blocked with 1% BSA for 1 h and a solution of 10 µg mL^−1^ recombinant mouse ICAM‐1 with poly‐histidine tag(Cat. 50440‐M08H, SinoBiological) was injected to the supported lipid bilayer, incubated at RT for 40 min and tethered to 18:1 DGS‐NTA(Ni) through chelation. Tethered SLB was washed excessively with 1X PBS before use. For the immobilization of ICAM‐1, 10 µg mL^−1^ recombinant protein A (Cat.101100, Thermo Fisher) in 1X PBS was injected into detergent‐cleaned and dried glass coverslip, incubated for 30 min, washed with 1X PBS, blocked with 1% BSA for 1 h, before 10 µg mL^−1^ recombinant mouse ICAM‐1 with Fc‐tag (Cat. 796‐IC, R&D systems) was injected and incubated at RT for 40 min. The resulting substrate was then washed with 1X PBS before use. For ECM protein coating on substrates, cleaned coverslips were incubated with 1 mg mL^−1^ collagen or 10 µg mL^−1^ fibronectin for 1 h at RT, and rinsed with PBS before use.

*Shear Flow and Adhesion Analysis*: The microfluidic device was created in‐house using a micromilling platform, design and fabrication protocols, and soft‐lithography techniques for shear flow and adhesion analysis.^[^ ^23^ ^]^ Within each device, SLBs were formed in two geometrically identical (mirrored), parallel microfluidic channels separated by a 250 µm barrier. The design and toolpaths for the double channel microdevice (channel height 1 mm, channel width 2 mm, length 16 mm) were created in Autodesk Fusion 360 (San Rafael, CA) and custom‐milled (Shapeoko, Carbide 3D, Torrance, CA) out of polycarbonate. The final device was manufactured by pouring polydimethylsiloxane (PDMS) mixed at a 10:1 base‐to‐curing agent ratio (Sylgard 184 elastomer kit; Dow Corning). PDMS was cured at 80 °C for 3 h, peeled off, and cut into individual devices. Channel inlets and outlets with 0.75 mm diameter were punched at both ends of microfluidic channels. The PDMS devices were permanently bound to the detergent‐cleaned glass coverslips after plasma treatment for 50 s (Harrick Plasma, Model PDC‐001‐HP) for the subsequent lipid bilayer formation and substrate modification.

A dual‐channel syringe pump (New Era Pump Systems, NY) was used to apply controlled shear flow to the two channels through 10 mL glass syringes (inner diameter 14.57 mm) and tubing connections. Cells were labeled with Calcein‐AM (Cat. C1430, Thermo Fisher) following the vendor's instructions, and incubated with substrates for 1 h under hypoxia conditions (37 °C, 4% CO_2_, and 5% O_2_), before infusing serum‐free RPMI 1640 media at controlled flow rates (ramping up from 0 to 30 mL min^−1^, with 10 s holding of each flow rates in a stepwise fashion) under a 37 °C environment. The design enables real‐time imaging and direct comparison of two cell types on the same substrate under the same flow rates. BF images were taken once every second using a 2x objective (CFI60 Plan Apochromat Lambda Lens, NA 0.1, WD 8.5 mm). The remaining cells under each flow rate were normalized as a percentage by the starting cell numbers in the same regions of interest (ROIs). Each ROI is a 500 × 500 µm square containing 20–80 cells randomly selected along the center of the channel. Shear stress at the SLB surface (bottom of the channel) was calculated at https://www.elveflow.com‐/microfluidic‐calculator/, where the fluidic properties were assumed the same as water at 37 °C considering the serum‐free nature of the RPMI 1640 media.

### In Vitro Migration and Invasion Assay

Boyden chamber experiments were conducted using 8‐micron transwell inserts. In brief, cells were serum‐starved for 24‐h prior to staining with a live‐cell dye (CellTracker Green) and seeding at a density of 5 × 10^4^ cells in the upper chamber in serum‐free media. Chemoattractant (10% FBS) was added to the bottom chamber and cells were allowed to migrate across the membrane for 18 h. After brief fixation, cells attached to the top of the insert were gently removed after fixation and the insert with cells migrated to the bottom of the insert were mounted on slides, imaged, and counted using the Keyence BZ‐X810 microscope quantified in the BZ‐X800 Analyzer Software. Invasion assays were performed in the same way, except using inserts that were purchased pre‐coated in Matrigel (Corning).

### In Vivo Experiments

Orthotopic tumors were established by mammary fat pad injections into 6–8 weeks old female NSG mice. Mice were given analgesic (Ketoprofen 5 mg kg^−1^) and general anesthesia (2% isoflurane) and placed in supine position on a heating pad with limbs immobilized. The fur around the fourth mammary gland on the mouse's right abdomen was shaved and disinfected with three alternating scrubs of chlorhexidine/iodine and sterile alcohol prep pads. A surgical incision was made medial to the fourth nipple in the abdominal skin and 100 µL of tumor cells were suspended in a 1:1 mixture of PBS:Matrigel was slowly injected into the fat pad. The wound was closed and animals were monitored for at least 3 days post‐surgery to ensure recovery.

Lateral tail vein injections were performed on 6–8 weeks old female NSG mice. Mice were placed in heated chambers before restraint in order to dilate the tail veins for injection. After visualization of the lateral tail veins, tails were sterilized with an alcohol swab and 100 µL suspension of cells in PBS were injected using a 26 and 5/8th gauge needle. After injection, pressure was firmly applied to the injection site for at least 1 min to stop bleeding.

For all mouse experiments for which luminal, ER+ cell lines were used, an estrogen pellet was subcutaneously implanted. For doxycycline induction of hairpins in mice, doxycycline was administered in the drinking water at 1 mg mL^−1^ with 1% sucrose in sterile water and changed every 2–3 days. In vivo bioluminescent imaging was performed by intraperitoneal injection of 100 µL of D‐luciferin substrate and subsequent imaging on the IVIS Lumina III instrument (PerkinElmer).

Luciferase assay for measuring transendothelial cell migration was performed as previously described.^[^ ^16^ ^]^ Briefly, lungs were harvested 24 h after tail vein injection of 1 00 000 cells per mouse and snap‐frozen in liquid nitrogen. Frozen lungs were pulverized into a powder using a pre‐chilled mortar and pestle and weighed. Using the luciferase assay system (Promega), cells were mechanically and chemically lysed and luciferase activity was quantified per mg of tissue in a Lucetta Luminometer (Lonza) using a 2 s delay and 10 s integration of luciferase signal. Normal lung lysate and lysate with a defined number of spiked‐in cells were analyzed to establish a limit of detection (LOD; Figure S2I, Supporting Information), which was set as a threshold to define the presence of metastases. LOD was calculated with the formula: LOD = mean_blank_ + 1.635(SD_blank_) + 1.635(SD_low concentration sample_), where mean_blank_ and SD_blank_ are the mean and SD of the replicates of a blank sample, and SD_low concentration sample_ is the SD of the replicates of the sample containing the lowest concentration of the cell lysate.

The animal experiments' protocol was approved by the Institutional Animal Care and Use Committee of the University of Southern California, under protocol #21127. All experiments were conducted in accordance with this protocol.

### Immunofluorescence on Tissue Sections

At the experimental endpoint, mice were euthanized by CO2 asphyxiation and cervical dislocation before harvesting lungs. Lungs were briefly rinsed in ice‐cold PBS and placed in cold 4% paraformaldehyde for 4.5 h with rocking, followed by 3 × 10 min washes with cold PBS and cryoprotected in 30% sucrose solution in PBS at 4 °C overnight. The next day, tissues were embedded in OCT and stored at −80 °C prior to sectioning.

Cryosections were incubated at 4 °C overnight with antibodies against chicken anti‐GFP (1:2000 dilution, Abcam ab13970) and rabbit anti‐cleaved caspase 3 (1:400 dilution, Cell Signaling Technologies 9661). The next day, secondary antibodies goat anti‐chicken IgY Alexa Fluor 488 (1:500 dilution, Life Technologies A11039) and goat anti‐rabbit IgG Alexa Fluor 647 (1:500 dilution, Life Technologies A32733) were incubated for 1 h at room temperature. Images were taken on a Keyence BZ‐X810 microscope and the signal was quantified in the BZ‐X800 Analyzer Software.

## Conflict of Interest

The authors declare no conflict of interest.

## Author Contributions

D.S.K. and A.M. contributed equally to this work. D.S.K., A.M., and M.Y. conceived the study, designed and performed experiments, and contributed to the analysis and interpretation of data. Y.A. and A.T. conducted computational analyses. D.S.K. and S.A. conducted MPA studies. B.U. and K.X. conducted super‐resolution imaging. J.H. and K.S. performed and interpreted the microfluidic adhesion study. D.S.K., A.M., A.T., and M.Y. wrote the manuscript. All authors edited or commented on the manuscript. M.Y. supervised the study.

## Supporting information

Supporting Information

## Data Availability

The data that support the findings of this study are available from the corresponding author upon reasonable request.
