# Supplementary material for: Ectopic Expression of a Truncated Isoform of Hair Keratin 81 in Breast Cancer Alters Biophysical Characteristics to Promote Metastatic Propensity
Source: Adv Sci (Weinh). 2023 Nov 10;11(5):2300509. doi: 10.1002/advs.202300509 (PMC10837353; doi:10.1002/advs.202300509)
Supplement: Supplementary file 1 — Supporting Information [file ADVS-11-2300509-s001.pdf]

ADVANCED SCIENCE
Open Access

Supporting Information

for *Adv. Sci.*, DOI 10.1002/advs.202300509

Ectopic Expression of a Truncated Isoform of Hair Keratin 81 in Breast Cancer Alters Biophysical Characteristics to Promote Metastatic Propensity

*Diane S. Kang, Aidan Moriarty, Yiru Jess Wang, Amal Thomas, Jia Hao, Bret A. Unger, Remi Klotz, Shamim Ahmmed, Yonatan Amzaleg, Stuart Martin, Siva Vanapalli, Ke Xu, Andrew Smith, Keyue Shen and Min Yu**

Supplementary Figures

A

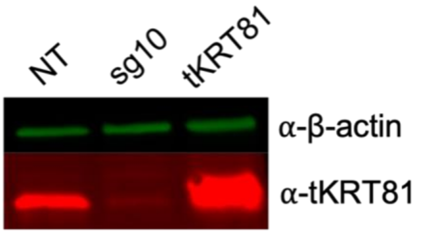

B

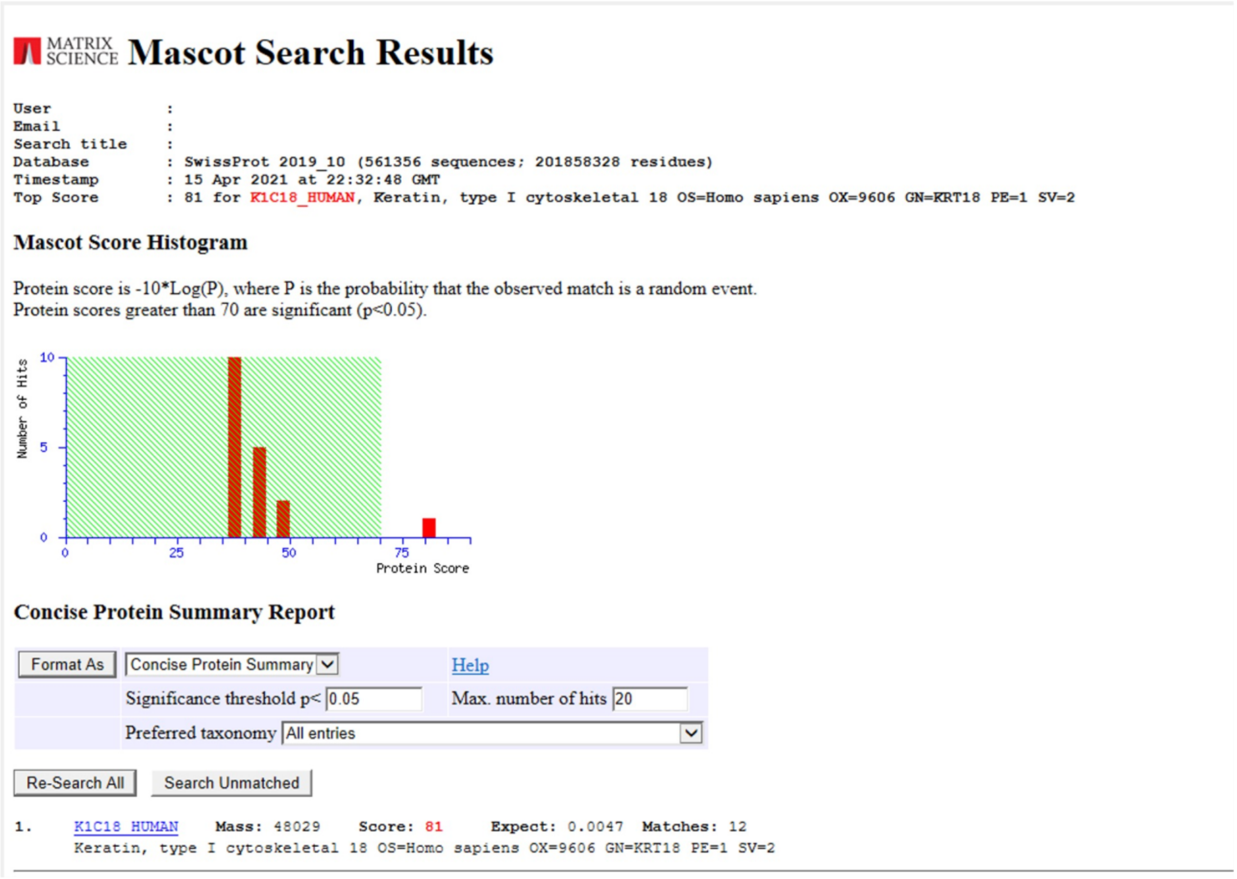

Figure S1. Mass spectrometry identifies physical interaction between KRT18 and tKRT81. **(A)** Immunoblot analysis of tKRT81 knockdown by CRISPRi (sg10) and overexpression (tKRT81) compared to control (NT) in MDA-MB-361 cells. β-actin is used as a loading control. **(B)** Immunoprecipitation of complexes binding to ectopically expressed tKRT81-DDK and mass spectrometry confirmed that KRT18 physically interacts with tKRT81.

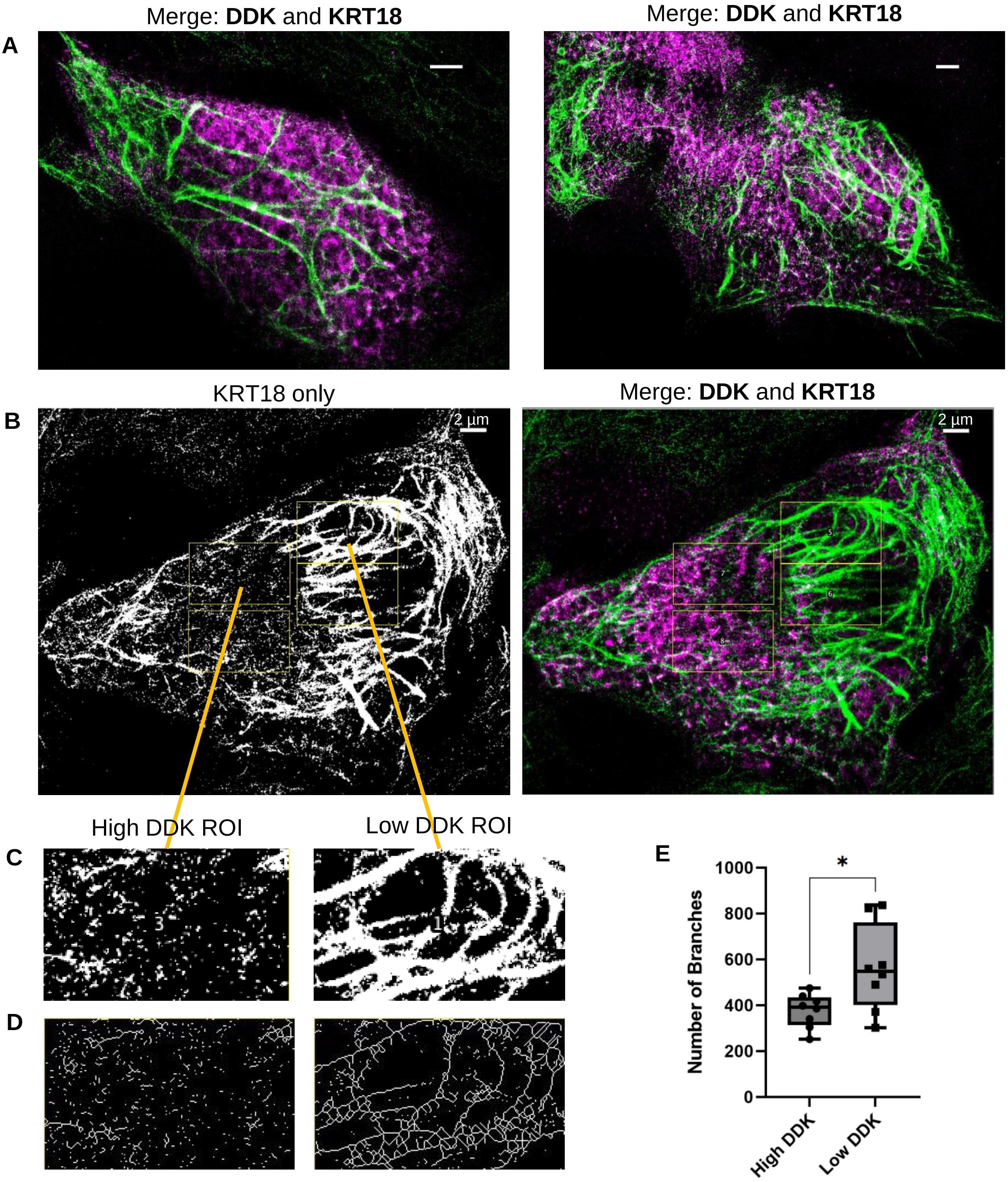

Figure S2. Quantification of cytoskeletal integrity in tKRT81-DDK expressing MDA-MB-361 cells shows higher expression is associated with lower keratin 18 integrity.
(A) Additional representative images of STORM super resolution images showing DDK (pink, tKRT81) and KRT18 (green) expression. **(B)** Representative binarized image of KRT18 channel (left) from composite STORM super resolution image of DDK and KRT18 (right) used for quantification analysis. **(C)** Example of extracted ROIs from KRT18 image for High DDK (left) and Low DDK (right) ROIs. **(D)** Example of skeletonized image from binarized ROIs created using Skeleton plugin in Fiji. **(E)** Quantification of the number of branches from High and Low DDK ROIs as metric for keratin integrity using Skeleton plugin. Mean ± SEM, n= 8 ROI per condition. **$p<0.0023$, significance calculated by unpaired t-test

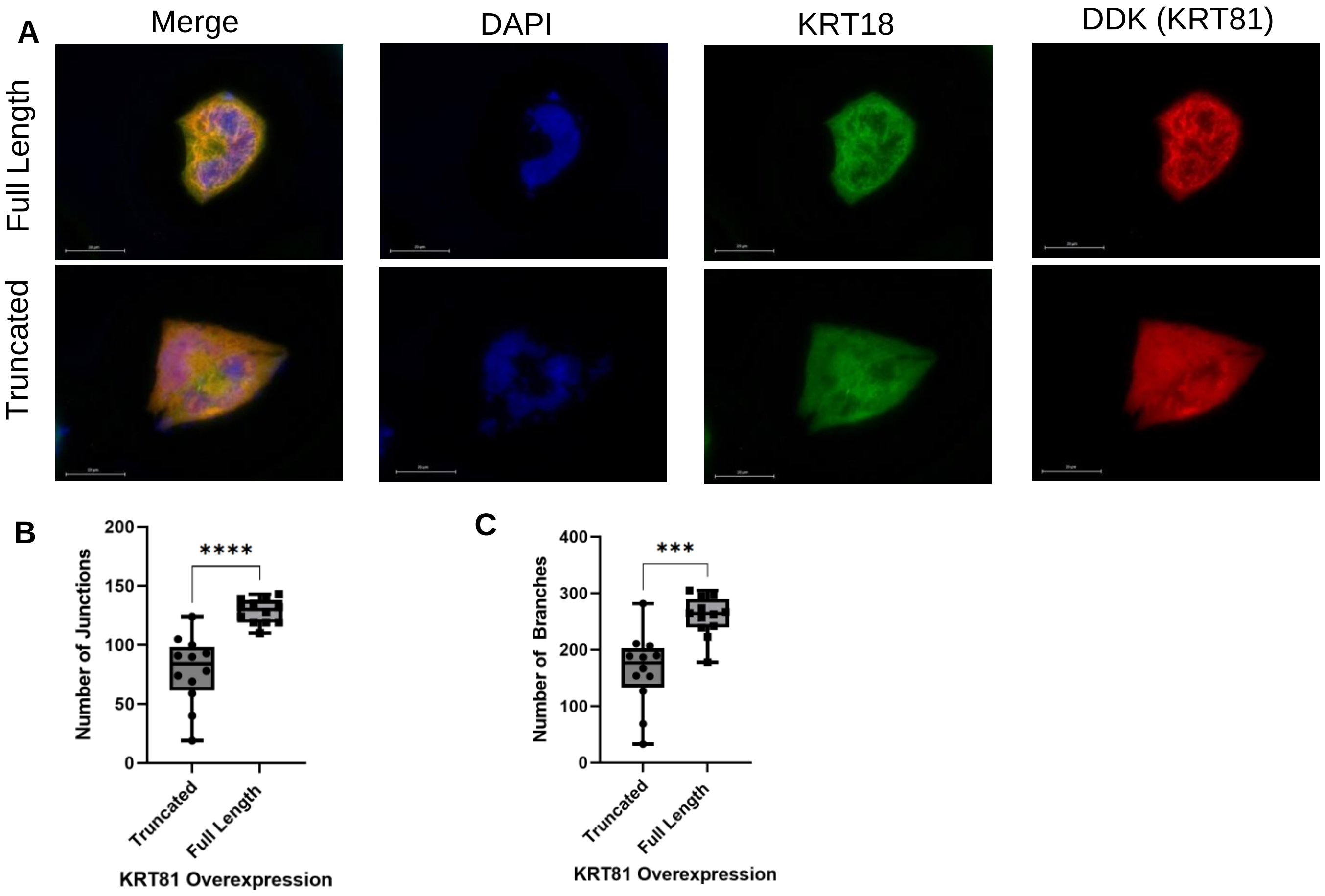

Figure S3. KRT18 bundle integrity is disrupted in tKRT81 but not full-length-KRT81 expressing MDA-MB-361 cells.

(A) Representative images of tKRT81 knockdown cells transiently transfected with either full length KRT81-DDK (top) or truncated KRT81-DDK (bottom). All images taken at 100x magnification. **(B)** Quantification of KRT18 filament integrity from ROIs of cells transiently transfected with full length or truncated KRT81 measured by the mean density of junctions using the Skeleton plugin in Fiji. Mean ± SEM, n= 12. ****$p<0.0001$, statistical significance calculated by unpaired t-test. **(C)** Quantification of KRT18 filament integrity from ROIs of cells transiently transfected with full length or truncated KRT81 measured by the number of branches using the Skeleton plugin in Fiji. Mean ± SEM, n= 12. ***$p<0.0002$, statistical significance calculated by unpaired t-test.

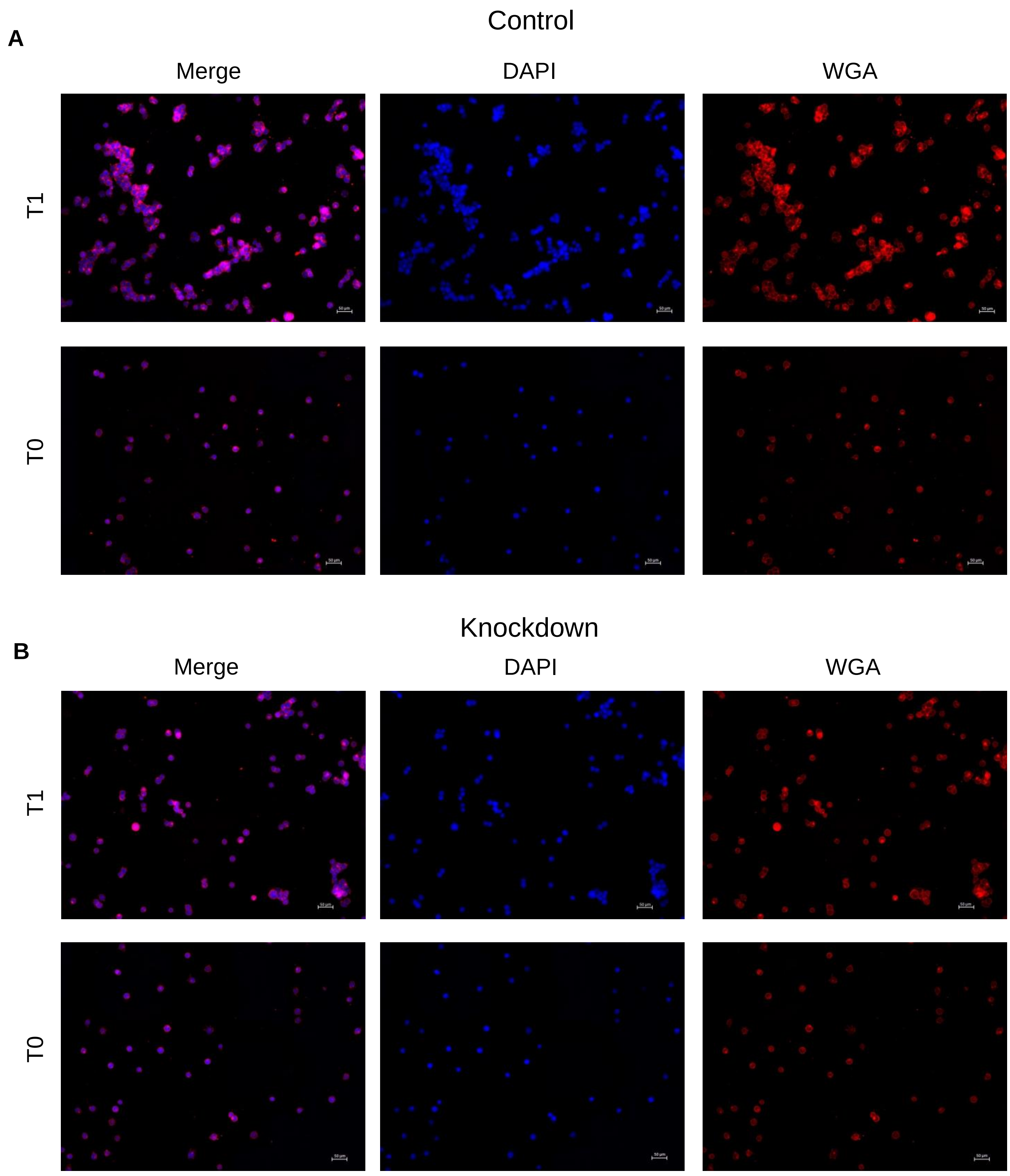

Figure S4. Efficiency and size of clusters formed is reduced with knockdown of tKRT81. (A-B) Representative images of cluster formation at 1 hour (top, T1) compared to time 0 (bottom, T0) in MDA-MB-361 control and tKRT81 knockdown cells. DAPI was used as a nuclear stain, and Wheat Germ Albumin (WGA) was used as a cell membrane stain. All images acquired at 10x magnification. **(A)** Images of cluster formation in tKRT81 expressing control cells at T1 (top) and T0 (bottom). **(B)** Images of clyster formation in tKRT81 knockdown cells at T1 (top) and T0 (bottom)

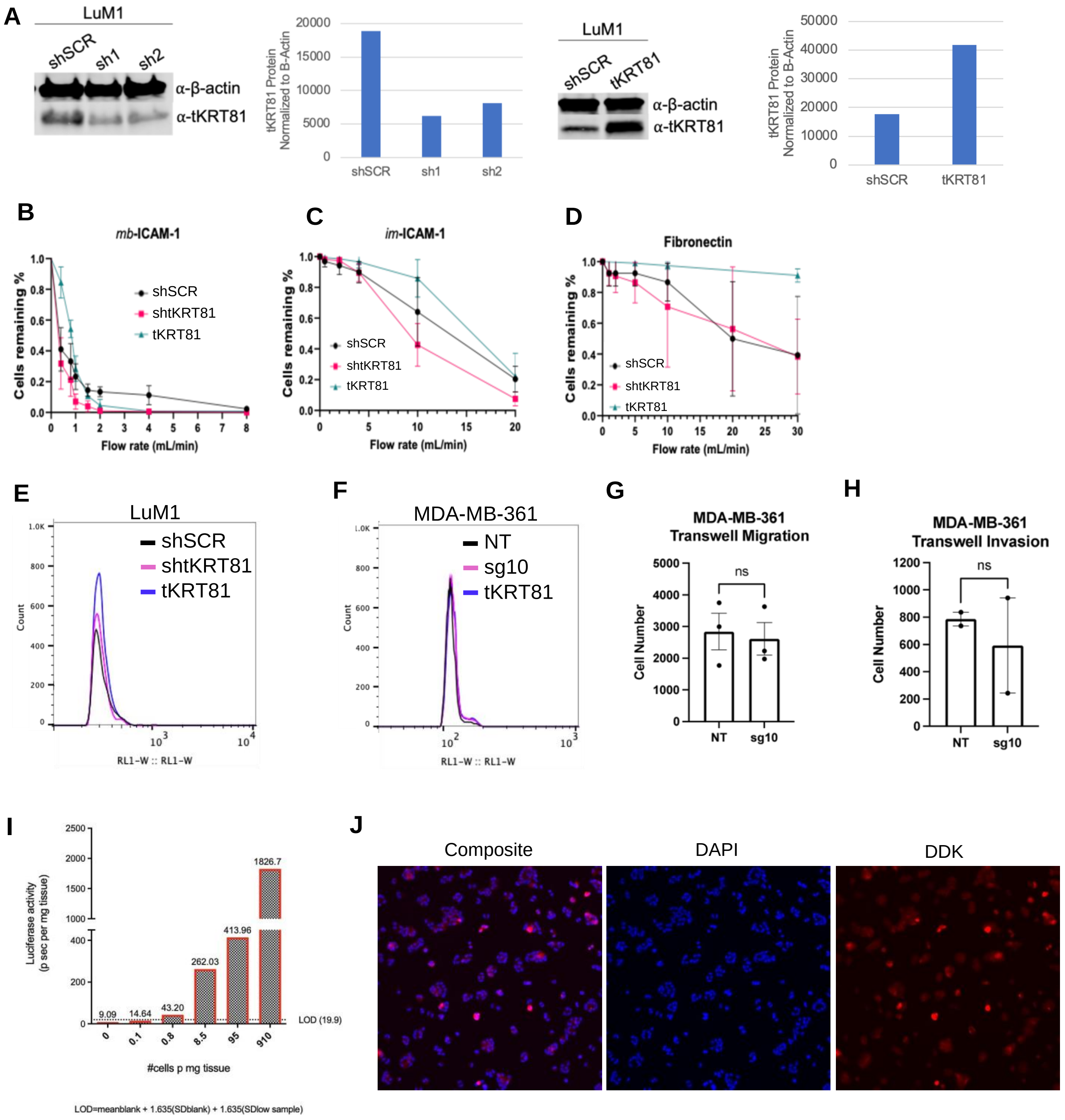
A
LuM1
shSCR
sh1
sh2
α-β-actin
α-tKRT81
tKRT81 Protein Normalized to B-Actin
20000
15000
10000
5000
0
shSCR
sh1
sh2
LuM1
shSCR
tKRT81
α-β-actin
α-tKRT81
tKRT81 Protein Normalized to B-Actin
50000
40000
30000
20000
10000
0
shSCR
tKRT81
B
mb-ICAM-1
Cells remaining %
shSCR
shtKRT81
tKRT81
Flow rate (mL/min)
C
im-ICAM-1
Cells remaining %
shSCR
shtKRT81
tKRT81
Flow rate (mL/min)
D
Fibronectin
Cells remaining %
shSCR
shtKRT81
tKRT81
Flow rate (mL/min)
E
LuM1
shSCR
shtKRT81
tKRT81
Count
RL1-W :: RL1-W
F
MDA-MB-361
NT
sg10
tKRT81
Count
RL1-W :: RL1-W
G
MDA-MB-361
Transwell Migration
ns
Cell Number
NT
sg10
H
MDA-MB-361
Transwell Invasion
ns
Cell Number
NT
sg10
I
Luciferase activity (p sec per mg tissue)
9.09
14.64
43.20
262.03
413.96
1826.7
LOD (19.9)
#cells p mg tissue
LOD=meanblank + 1.635(SDblank) + 1.635(SDlow sample)
J
Composite
DAPI
DDK

Figure S5. tKRT81 expression does not affect cell adhesion to ICAM and fibronectin, nor impact on cell migration and invasion

(A) Immunoblot analysis of tKRT81 knockdown by shRNAs (sh1 and sh2) and overexpression (tKRT81) compared to control (shSCR) in LuM1 cells. β-actin is used as a loading control. Quantification of tKRT81 protein shown to the right of the repspective immunoblots was determined by normalizing protein levels of tKRT81 to beta-actin normalized across samples within a blot. **(B-D)** Graphs showing the percentage of cells remain adhered to membrane bound ICAM **(B)**, immobilized ICAM **(C)**, or fibronectin **(D)** coated channels after application of shear force with increasing flow rates for LuM1 control (shSCR), tKRT81 knockdown (shtKRT81), and tKRT81 rescue overexpression (tKRT81) cells. **(E-F)** Histograms showing flow cytometry analysis of cell surface localization and expression of integrin β1 in LuM1 tKRT81-expressing control (shSCR), tKRT81 knockdown (shtKRT81), and overexpression (tKRT81) cells **(E)** and MDA-MB-361 control (NT), tKRT81 knockdown (sg10), and overexpression (tKRT81) cells **(F)**. (**G-H)** Bar graphs showing number of cells on the bottom of the transwell membrane in transwell migration **(G)** for MDA-MB-361 control (NT) and tKRT81 knockdown (sg10) cells and transwell invasion **(H)** assay for the same cell lines. Mean ± SEM. **(I)** Bar graph showing the establishment of limit of detection (LOD) for the *in vivo* transendothelial cell migration assay by *ex vivo* luciferase activity assay. **(J)** Representative images demonstrating the heterogeneity of tKRT81-DDK expression in the MDA-MB-361 tKR81-DDK overexpressing rescue cell line. An anti-DDK antibody was used to visualize tKRT81-DDK expression. All images taken at 4x magnification.

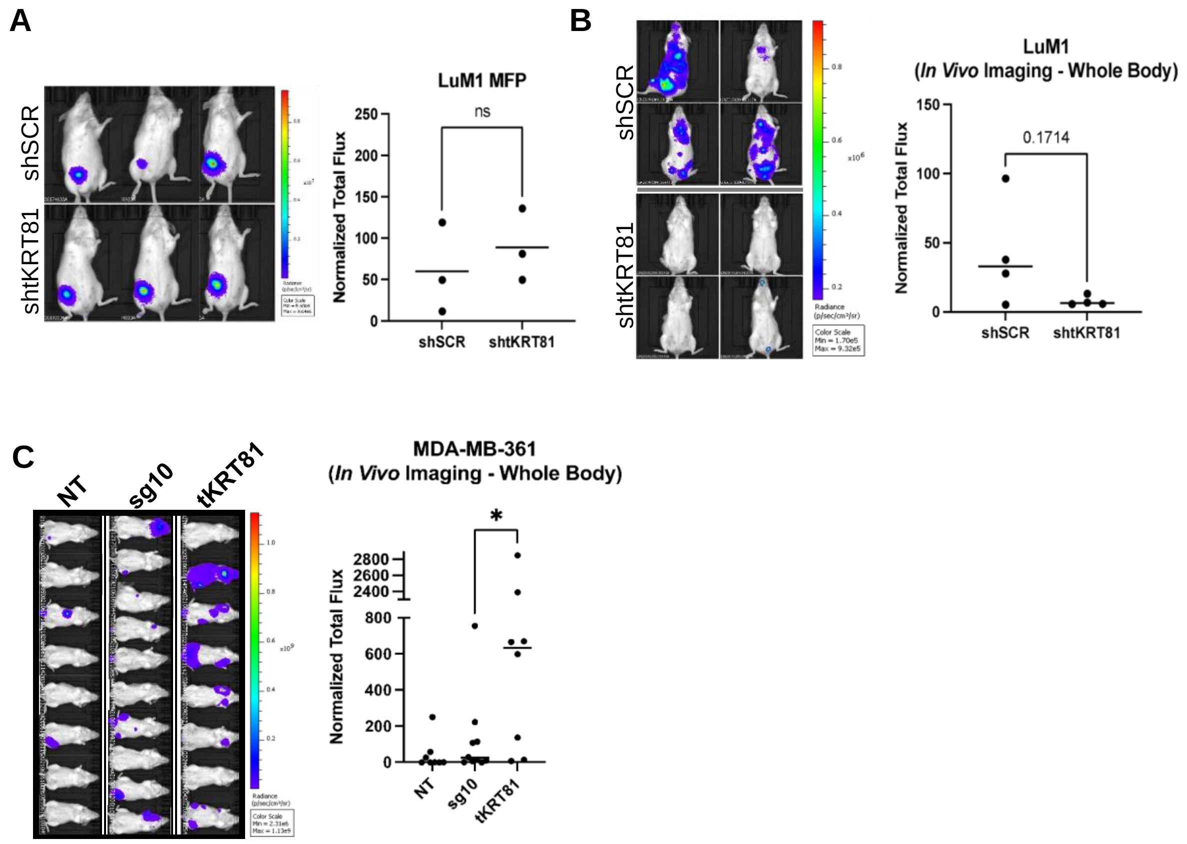

Figure S6. Expression of tKRT81 has no significant *in vivo* consequence on tumor growth at orthotopic site or lung metastasis in some cell lines

(A) Bioluminescence imaging (left) and quantification (right) of orthotopic tumor growth of LuM1 control (shSCR) and tKRT81 knockdown (shtKRT81) cells injected into mammary fat pad of NSG mice. **(B)** Whole body bioluminescence imaging (left) and quantification (right) of NSG mice with tail vein injection of LuM1 control (shSCR) and tKRT81 knockdown (shtKRT81) cells. **(C)** Whole body bioluminescence imaging (left) and quantification (right) of NSG mice with tail vein injection of MDA-MB-361 control (NT), tKRT81 knockdown (sg10), and tKRT81 overexpression rescue (tKRT81) cells. Mean ± SEM. *p<0.05, significance is calculated by unpaired t-test.
